# Whole-body MRI in arthritis

**DOI:** 10.1007/s00256-025-04998-z

**Published:** 2025-08-29

**Authors:** Fabio Zecca, Denise Dejua, Winston J. Rennie, Luca Saba

**Affiliations:** 1 Department of Radiology, University Hospital of Cagliari, Cagliari, Italy; 2 Department of Radiology, Leicester Royal Infirmary, Leicester, United Kingdom; 3 Department of Medical Imaging, University Cancer Institute of Toulouse – Oncopole, Toulouse, France

**Keywords:** Inflammatory arthritis, Juvenile idiopathic arthritis, Rheumatoid arthritis, Spondyloarthropathy, Whole-body imaging

## Abstract

Whole-body MRI (wbMRI) is an advanced scan modality which provides high-contrast resolution images of the entire body for screening, diagnosis, staging, and surveillance purposes. Thanks to recent technological advancements, wbMRI has gained increasing attention as a valuable tool for diagnosing and monitoring systemic arthritides by virtue of its comprehensive coverage. This technology is particularly beneficial for inflammatory conditions like rheumatoid arthritis, seronegative spondyloarthropathy, juvenile idiopathic arthritis, and chronic aseptic osteitis, all characterized by multifocal involvement of both skeletal and extra-skeletal sites. Unlike conventional MRI targeting symptomatic areas, wbMRI enables concurrent early detection also of subclinical disease foci, providing a more accurate assessment of the total disease burden. Compared to plain radiography, conventional ultrasound, and targeted MRI, wbMRI offers better sensitivity and reproducibility, particularly for intraosseous findings and axial involvement. In addition, wbMRI can be tailored to specific clinical needs through customizable scan protocols, and the lack of ionizing radiation makes it ideal for monitoring autoimmune diseases also in sensitive cohorts. Although wbMRI holds great potential for improving diagnostic accuracy and patient outcomes in inflammatory arthritis, further research is needed to validate standard scan protocols, to confirm their cost-effectiveness, and to integrate them into routine clinical practice. The present article aims to overview the current wbMRI technology and discuss it in the context of inflammatory arthritis. A general wbMRI protocol for non-oncologic musculoskeletal application is also provided.

## Introduction

Arthritis represents a heterogeneous group of diseases variably leading to painful functional loss, skeletal deformity, and impaired life quality in several age groups [1–3]. Most autoimmune forms like rheumatoid arthritis (RA) and seronegative spondyloarthropathy (SpA) prefer the adult population, while others such as juvenile idiopathic arthritis (JIA) selectively affect children [4, 5]. To this add the osteoarticular manifestations of the chronic aseptic osteitis (CAO) spectrum, whose synovitis, acne, pustulosis, hyperostosis, and osteitis (SAPHO) syndrome and chronic recurrent bacterial osteomyelitis (CRMO) are oftentimes regarded as rare SpA variants [6, 7]. Notably, several non-primarily musculoskeletal autoimmune conditions such as systemic lupus erythematosus, systemic sclerosis, and Sjögren syndrome can be variably accompanied by joint inflammation [8].

Whereas clinical presentation and laboratory tests are often sufficient for differentiating among the main types of arthritis, imaging is essential for reliably assigning each patient to specific subgroups with different management and prognosis. Although plain radiography (XR) is still the recommended first-line imaging technique for most joint pathologies, magnetic resonance imaging (MRI) represents the most convenient modality, enabling earlier detection, accurate diagnosis, and treatment monitoring in a safe, tailorable, and reproducible manner [3, 9]. Nevertheless, conventional “targeted” MRI scans can miss possible subclinical inflammation foci, preventing a comprehensive disease overview valuable for diagnostic and prognostic purposes [10]. Whole-body MRI (wbMRI) is an advanced scan modality that has reached clinical applicability for diverse oncologic and non-oncologic affections [9, 11, 12]. In fact, the broad anatomic coverage of wbMRI overcomes the limits of conventional MRI acquisitions, easily capturing multifocal inflammation in both skeletal and extraskeletal sites [4, 8, 10]. To date, wbMRI boasts a promising profile in several rheumatologic affections, including RA [13], SpA [14], and JIA [15].

The present review is composed of three sections. The first section offers a comprehensive overview of the technical aspects, strengths, and limitations of current wbMRI technology. The second section discusses the role of wbMRI in the context of several inflammatory arthritides, including a concise overview of the characteristics, imaging, and guidelines for each. The third section provides a general wbMRI protocol for non-oncologic musculoskeletal application, along with elements to take into account according to the concerned pathology.

## Whole-body MRI: overview

The rise of the whole-body potential of MRI can be traced back to the 1990s, when multiple receiver coils and sliding scanner tables were firstly introduced. Later technological advancements in terms of magnetic field quality and sequence design paved the way for the current wbMRI applications in research and clinical practice [9, 16]. The current wbMRI technology boasts excellent tissue contrast capabilities and good spatial resolution, potentially contributed by supplementary functional data. All such information gets extracted from a very large field of view (FoV) while bypassing the disadvantages of ionizing radiation, contrast agents, and radioactive tracers. While conventional smaller-FoV MRI acquisitions targeted on symptomatic regions may miss asymptomatic disease foci, wbMRI overcomes such limitation by virtue of its broad anatomic coverage [9, 16, 17]. To date, the role of wbMRI has been extensively evaluated for several types of cancers [12, 17], and its profile has collected promising results also in the context of non-oncological conditions [9, 18]. Building on all the above advantages, wbMRI has steadily expanded also in the domain of pediatric radiology [19–21]. According to an ESSR survey (2018), RA, SpA, and SAPHO are among the most common non-oncological indications for wbMRI in adults after inflammatory myopathies, while CRMO is the most common pediatric indication. In the context of systemic inflammation syndromes, wbMRI is mostly utilized for diagnosis, lesion mapping, and monitoring, often directing patient management [5].

**Fig. 1:**
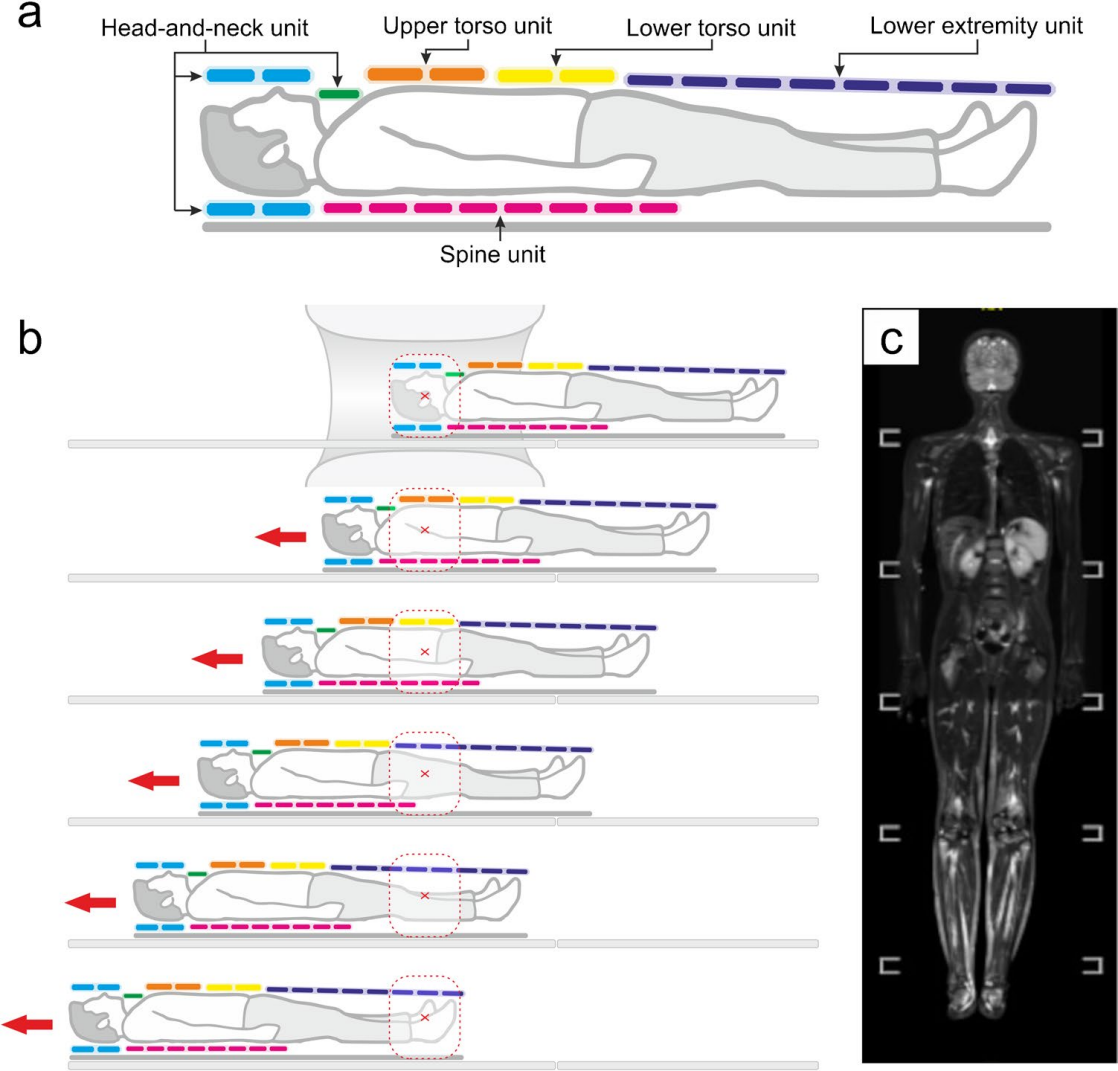
Whole-body MRI setup, acquisition, and coverage. **a** Schematic showing a typical coil setup; **b** Schematic of the sequential scan process. Different body sections are isocentered and scanned in separate steps or “stations” during table movement (red arrows); **c** Example of a stitched coronal STIR image, showing the comprehensive coverage from vertex to toes in the coronal view. Active inflammation in both knees is notable (panel **c**: kind courtesy of Jurik AG, spa-imaging.org)

By default, the wording “whole-body MRI” refers to a series of contiguous vertex-to-toes coronal images acquired sequentially across multiple “stations” of 25–50 cm each and stitched together during post-processing (Fig. 1). Owing to the time constraints of daily practice, wbMRI protocols get frequently optimized to the clinical question in terms of pulse sequences and body coverage. On average, the total scan time of a wbMRI exam ranges between 45 and 75 minutes plus a setup time of 5–10 minutes, collectively fitting examination slots of 60–90 minutes each [9, 16, 17]. Truncation to narrower coverage (e.g., orbits-to-knees, shoulders-to-hips) can thus be convenient in certain scenarios although requiring clear annotation (e.g., “CAP wbMRI” for chest, abdomen, and pelvis; “NCAPPE wbMRI” for neck, chest, abdomen, pelvis, and proximal extremities) and completion via regional scans of the neglected body parts whether necessary. Moreover, as the large-FoV advantages are counterbalanced by reduced spatial and contrast resolution, whole-body acquisitions may benefit from ancillary smaller-FoV acquisitions targeted to susceptible sites, for better depicting subtle abnormalities and ultimately preserving diagnostic reliability [4, 9, 16, 17, 22]. In standard wbMRI acquisitions, the patient is lying supine with the upper limbs resting along the body, ideally with the palms parallel to the sagittal plane and the soles orthogonal to the coronal plane (Fig. 1). Alternatively, the hands can be positioned on the belly or behind the buttocks, which is often necessary in the case of larger patients for keeping their upper limbs within the FoV [17, 23, 24].

**Fig. 2:**
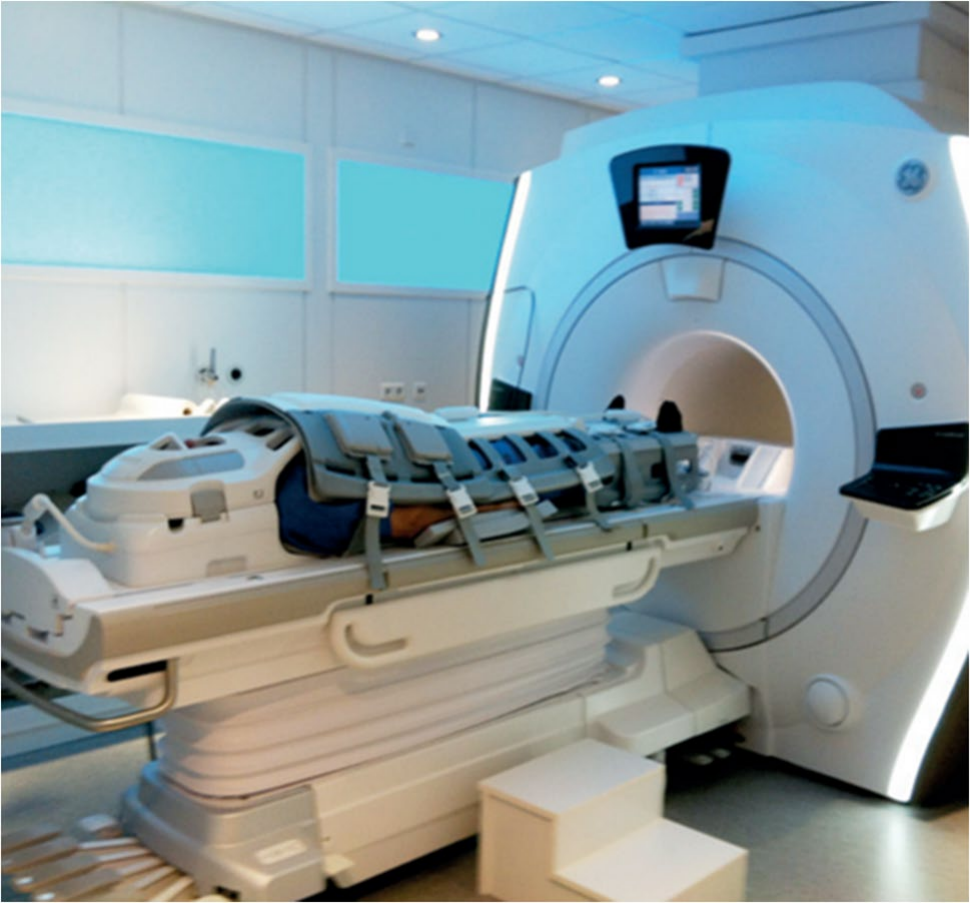
Example of a whole-body MRI exam performed using a static composite coil setup (image reproduced from Tárnoki et al., 2015 [138], originally licensed under CC BY-NC-ND 3.0)

Whole-body coverage in wbMRI is generally obtained by acquiring two to seven stations, the exact amount depending on the patient’s height as well as the scanner’s FoV along the *z*-axis. This “multistation imaging” technique requires the scanner table to manually or automatically move relative to the magnetic isocenter. Established whole-body coverage strategies rely either on static composite coil setups (e.g., Total Imaging Matrix (TIM), Siemens; dStream WholeBody coil, Philips) or moving surface coils (e.g., Angiographic System for Unlimited Rolling Fields-of-view (AngioSURF), MR-Innovation GmbH; Stepping Kinematic Imaging Platform (SKIP), Magnetic Moments), while current innovation efforts are focusing on special whole-body volume coils and customized printable coil elements [9, 16, 17, 25–29]. Nowadays, the most commonly employed strategy is likely represented by static composite coil setups mounted on automatically moving tables often relying on a head-and-neck integrated unit, an in-table spine unit, and several surface phased-array coils for the anterior torso and extremities (Figs. 1, 2). Conventional stepwise acquisition will be likely challenged by continuous acquisition via table sliding, with potential advantages in terms of image homogeneity and scan time possibly limited by stair-step artifacts and large data volume [9, 17]. High-field MRI scanners can offer higher signal-to-noise ratio (SNR) at the price of increased sensitivity to artifacts from body and organ movements, metallic material, and static-field inhomogeneity, nonetheless mitigable via slice-specific shimming and post-processing algorithms. Anyhow, reliable comparative data on the performance of 1.5-T vs. 3.0-T scanners for wbMRI are still missing [16, 17]. Wide-bore scanners can fit larger patients and decrease psychological distress, thus potentially preserving scan, quality although requiring more scan stations for achieving whole-body coverage. Open scanners can also be utilized, although burdened by poorer image quality and lower availability [17].

Modern wbMRI protocols mainly rely on STIR/TIRM sequences and/or DWI with ADC mapping, variably accompanied by T1w GRE, fat-saturated (FS) T2w TSE, and contrast-enhanced acquisitions. Moreover, multiplanar reformation (MPR) enabled by isotropic 3D acquisitions leaves full navigation control to the radiologist, enhancing diagnostic precision and improving workflow efficiency. Among conventional sequences, STIR boasts additive-contrast properties yielding excellent sensitivity for the fluid content seen in cellulated and cystic lesions, third-space effusions, and reactive tissue changes. In the context of rheumatological diseases, STIR is optimal for detecting edema in the bone marrow, ligaments, tendons, and juxta-articular tissues, as well as effusions in joints and bursae. Spoiled 3D T1w GRE sequences (e.g., VIBE) with Dixon chemical-shift acquisition yield water- and fat-selective images useful for qualitative analysis of the anatomical context. Ultra-fast T2w SS EPI sequences (e.g., HASTE) with chemical-shift spectral saturation (CHESS) of fat can provide additional information concerning the status of the spinal canal and other extraskeletal sites. Intermediate-weighted cartilage-oriented sequences can further contribute to joint assessment in specific scenarios [9, 30–33].

Furthermore, functional sequences such as SS EPI DWI sequences with ADC mapping can help to detect regions with restricted water diffusion such as densely cellular lesions, while the T2 shine-through effect can support the interpretation of whole-body DWI scans for inflammatory affections, even surpassing STIR [9, 16, 34, 35]. In whole-body DWI with background suppression (DWIBS), free breathing is intentionally allowed as the coherent cyclicity of physiological organ movements can blur the background leaving the diffusion-dependant signal relatively untouched. Although originally conceived for cancer staging and surveillance, DWIBS could have a role in rheumatologic affections, especially when coupled with morphologic sequences [36]. Regional SSFP T2*w GRE (e.g., FISP, PSIF, CISS, DESS) could also be of value in wbMRI for certain systemic affections by virtue of their tailorable final image contrast for solid organs, bowel, and lungs [9]. Supplementary UTE/ZTE acquisitions could enable the assessment of the lungs, compact bone, and connective tissues, while FLAIR sequences could detect further pathologic changes in the central nervous system [9].

Although rarely implemented in the context of wbMRI, intravenous gadolinium-based contrast agents (GBCAs) in complementary small-FoV scans can enhance hyperemic tissues and hypervascular lesions. Dynamic contrast enhancement (DCE) could further improve the diagnostic utility of enhanced wbMRI scans, although its implementation in whole-body acquisitions is impractical thus reserved to targeted small-FoV acquisitions. Nonetheless, routine use of intravenous GBCAs is not recommended in case of non-infectious inflammatory conditions [9, 17]. Moreover, ultra-small paramagnetic iron oxide (USPIO) particles could provide information about the status of the lymphoreticular system [9]. Hybrid PET-MRI systems can simultaneously extract various morphologic and functional information of diagnostic and prognostic value from musculoskeletal tissues. However, the actual cost-effectiveness of whole-body PET-MRI for non-oncologic musculoskeletal affections in the clinical setting still needs further investigation [37].

Generally, T1w sequences do require breath holding while optimized T2w sequences can also be executed in free breathing. Respiratory and cardiac triggering are necessary to minimize pulsation and breathing artifacts in the thoracic and upper abdominal regions. DWI should utilize several averages for reducing motion artifacts and increasing SNR and a single diffusion encoding direction with triaxial gradients for further increasing SNR while reducing scan time. A low *b*-value > 50 s/mm^2^ and high *b*-values > 800 s/mm^2^ are commonly employed for revealing microvascular contribution to the DWI signal and improving detection of abnormalities [9, 16]. Synthetic ADC values obtained via computed ultrahigh *b*-value (i.e., up to 2000 s/mm^2^) DWI could potentially improve the diagnostic accuracy of wbMRI studies while preserving scan conciseness, as already demonstrated in different contexts [38–40]. Whole-body acquisitions in the coronal plane ensure the lowest scan times by minimizing the number of slices, although coronal DWI images might suffer from artifactual distortion compared to axial images. The ideal balance between through-plane resolution and clinical time constraints in wbMRI implies a slice thickness of 5–7 mm with an interslice gap of 1 mm, although smaller-FoV musculoskeletal acquisitions could benefit from thinner slices of 2–3 mm interspaced with 0.3-mm gaps [16, 33, 41].

In addition to the general limitations of the MRI technology such as long scan times, limited availability, high costs, patient-related contraindications, and need for contrast agent in certain cases [33], current wbMRI technologies do face several challenges. Lack of dedicated coils, patient positioning issues, and larger voxels contribute to lower image quality compared to conventional MRI at the same field strength. Also, whereas in conventional MRI the hand is placed in a dedicated coil closer to the magnetic isocenter, in wbMRI the hands and forearms lie more off-center, particularly in larger individuals. Moreover, the relatively thick slices affect in-plane resolution in wbMRI, which hampers image interpretability especially for the small peripheral joints. Complementary targeted acquisitions of the upper limb can preserve the overall image quality at the price of longer scans, while positioning the hands on the belly or under the buttocks would preserve scan duration although at the price of lower image quality due to decentering [9, 13, 42]. Furthermore, the sole use of anisotropic coronal scans can limit the diagnostic accuracy for certain skeletal regions, such as the ribs and sternum. The watershed area between two scan stations can be affected by stitch artifacts, which can be minimized via phase oversampling and increased station overlap. Younger children commonly require sedation or general anesthesia, although hypnosis, mock MRI scanners, behavioral support, and audiovisual equipment represent alternative strategies worth attempting [9, 43–45].

## Whole-body MRI: applications in arthritis

### General aspects

The extreme sensitivity of MRI technology to musculoskeletal abnormalities has progressively built its pivotal role in rheumatologic diagnosis over the years. In fact, MRI has become the predominant outcome-measurement tool in rheumatology thanks to its diagnostic accuracy and reproducibility for what concerns inflammatory activity, structural damage, and response to treatment [13]. Notably, the OMERACT group has played a key role in promoting its utilization in rheumatologic research by developing several MRI-based scoring systems [42, 46, 47] (Table 1). In addition, societal collaborative works have provided guidance tools for assessing rheumatic lesions via MRI (Table 2) and reference atlases have also become available [48–50]. In general terms, fluid-sensitive sequences (e.g., T2w TSE FS/Dixon/STIR) and contrast-enhanced acquisitions (e.g., post-GBCA 3D T1w GRE FS/Dixon) acquisitions can provide hints concerning active osteoarticular inflammation, while higher-signal anatomical (e.g., PDw TSE FS, 3D T1w GRE Dixon) are useful for assessing established structural changes in the skeleton [42].

**Table 1:** Overview of the main pathologic entities encompassing inflammatory arthritis

Disease	Preferred population	Affected sites		MRI-based scoring systems	Notes
		Primary	Secondary		
RA	Adults, F > M	Peripheral skeleton (small joints)	Large peripheral joints, cervical spine, extraskeletal sites	RAMRIS, RAMRIS-5, SAMIS, ERAMRS	Mostly polyarticular and symmetrical; possibly monoarticular arthritis or palindromic rheumatism
axSpA	Young adults, M > F	Sacroiliac joints, thoracolumbar spine	Anterior thoracic joints, peripheral skeleton, extraskeletal sites	SPARCC, Berlin, ASspiMRI-a/-c, CANDEN	Includes AS; hallmarked by enthesitis; non-radiographic disease precedes radiographic changes
pSpA	Adults	Peripheral skeleton	Axial skeleton	HEMRIS, MRI-WIPE	Including most PsA, ReA, EnA, and uSpA cases
PsA	Adults	Peripheral skeleton, extraskeletal sites	Axial skeleton	PsAMRIS	Hallmarked by dactylitis (enthesitis-related, with diaphyseal osteitis and predominant involvement of flexor tendons); mostly asymmetrical; highly variable manifestations
ReA	Adults following infection, M > F	Lower extremities (large joints)	Upper extremities, axial skeleton	n/a	a.k.a. Reiter’s syndrome; mostly asymmetrical; triggered by infections; tends to chronicize; post-urethritis forms more severe MRI features than post-enteritis forms
EnA	IBD patients	Peripheral skeleton, extraskeletal sites	Axial skeleton	n/a	Associated with IBD and other chronic enteropathies; MRI might anticipate the diagnosis by highlighting concurrent arthritic and enteropathic features
uSpA	n/a	n/a	n/a	n/a	Exclusion diagnosis; often evolves into a more defined SpA form
JIA	Children	Peripheral skeleton	Temporomandibular joint, cervical spine	JAMRIS	Multiple subtypes, including jSpA forms; classification under renewal; early diagnosis critical for avoiding deformities; needs diagnostic familiarity with skeletal maturation
jSpA	Children, M > F	Sacroiliac joints, thoracolumbar spine	Peripheral skeleton, extraskeletal manifestations	n/a	Hallmarked by enthesitis; early asymmetrical peripheral involvement, possible later axial involvement;
SCCH	Adults	Anterior chest wall	Axial skeleton, peripheral skeleton	n/a	Incomplete SAPHO variant
PAO	Adults	Anterior chest wall	Axial skeleton, peripheral skeleton	n/a	Incomplete SAPHO variant; a.k.a., Sonozaki syndrome
SAPHO	Adults	Anterior chest wall, axial skeleton	Peripheral skeleton	n/a	Adult form of CRMO (?); hallmarked by synovitis, acne, pustulosis, hyperostosis, and osteitis
CNO	Children	Peripheral skeleton	Axial skeleton	n/a	Unifocal, milder form of CRMO
CRMO	Children	Peripheral skeleton	Axial skeleton	n/a	Pediatric form of SAPHO (?); hallmarked by multiple recurring inflammatory bone lesions

*AS* ankylosing spondylitis, *RA* rheumatoid arthritis, *JIA* juvenile idiopathic arthritis, *axSpA* axial spondyloarthritis, *pSpA* peripheral spondyloarthritis, *PsA* psoriatic arthritis, *ReA* reactive arthritis, *EnA* enthesitis-related arthritis, *uSpA* undifferentiated spondyloarthritis, *jSpA* juvenile spondyloarthritis, *CNO* chronic nonbacterial osteomyelitis, *SCCH* sternocostoclavicular hyperostosis, *PAO* pustulotic arthro-osteitis, *SAPHO* synovitis, acne, pustulosis, hyperostosis, osteitis, *CRMO* chronic recurrent multifocal osteomyelitis, *RAMRIS* Rheumatoid Arthritis Magnetic Resonance Imaging Score, *RAMRIS-5* Rheumatoid Arthritis Magnetic Resonance Imaging Score-5, *SAMIS* Spondyloarthritis Magnetic Resonance Imaging Score, *ERAMRS* Early Rheumatoid Arthritis Magnetic Resonance Imaging Score, *JAMRIS* Juvenile Arthritis Magnetic Resonance Imaging Score, *SPARCC* Spondyloarthritis Research Consortium of Canada, *ASspiMRI* Ankylosing Spondylitis Spine MRI, *CANDEN* Canada-Denmark, *SPARCC* Spondyloarthritis Research Consortium of Canada, *MRI-WIPE* MRI Whole-body score for Inflammation in Peripheral joints and Entheses, *PsAMRIS* Psoriatic Arthritis Magnetic Resonance Imaging Score

**Table 2:** Recently published societal guidelines concerning inflammatory arthritides, with a focus on MRI utilization

Disease	Society/group	Year	Document type	Main statements	Ref
RA	ACR*/EULAR	2010	Classification criteria	• RA can be diagnosed in patients with definite clinical synovitis in at least one joint no better explained by another disease whether scoring at least 6 out of 10 points across four domains (i.e., joint involvement, serology, acute-phase reactants, and symptom duration)	[134]
				• MRI considered for confirming joint involvement	
	EULAR	2013	Recommendations	• MRI recommended for early detection, diagnosis, monitoring, and prognostication	[135]
	ESSR Arthritis Subcommittee	2015	Recommendations	• MRI preferred for diagnosing and monitoring early disease, especially axial involvement	[33]
	ACR	2017	Appropriateness criteria	• MRI (or US) suggested as complementary imaging to XR in selected cases	[53]
axSpA	ASAS	2009	Classification criteria	• axSpA can be diagnosed in patients younger than 45 years with chronic back pain following either the “clinical arm” (i.e., positivity to HLA-B27 + ≥ 2 other SpA clinical features) or the “imaging arm” (i.e., imaging evidence of sacroiliitis + ≥ 1 SpA clinical feature)	[80–82]
				• MRI considered for confirming joint involvement	
	ASAS/OMERACT	2009	Definition criteria	• MRI-positivity for sacroiliitis: either one area of bone marrow edema on at least two consecutive slices or more than one area of bone marrow edema on a single slice	[82]
	ASAS/OMERACT	2011	Definition criteria	• MRI-positivity for spondylitis: anterior and posterior corner-based inflammatory lesions in three or more sites and subsequent fat metaplasia	[136]
	EULAR	2015	Recommendations	• MRI considered for predicting structural lesions and monitoring of disease activity	[79]
	ESSR Arthritis Subcommittee	2015	Recommendations	• MRI considered for initial assessment in selected cases, assessment of structural lesions, and monitoring of treatment response	[33]
	ACR	2021	Appropriateness criteria	• MRI suggested as complementary imaging to XR in selected cases, especially for monitoring treatment response	[54]
	ESSR Arthritis Subcommittee	2024	Reporting guidelines	• Axial MRI findings in axSpA (T1w SE, T2w TSE FS, and STIR at ≤ 1.5 T)	[85]
pSpA	ASAS	2011	Classification criteria	• pSpA can be diagnosed in patients with peripheral-only manifestations of arthritis, enthesitis, and/or dactylitis whether demonstrating other general SpA features	[99]
	EULAR	2015	Recommendations	• MRI (or US) considered for detecting peripheral inflammation, monitoring disease activity, and assessing structural changes	[79]
	ESSR Arthritis Subcommittee	2015	Recommendations	• MRI suggested in case of inconclusive XR and/or US for diagnosing inflammatory lesions, monitoring disease activity, and detecting complications (e.g., cartilage damage, tendon tears, avascular necrosis);	[33]
				• Synovial enhancement must be assessed within 10 min from injection before GBCA permeates into the synovial fluid	
	ACR	2017	Appropriateness criteria	• MRI (or US) suggested as complementary imaging to XR in selected cases	[53]
				• GBCA considered a powerful tool for identifying active inflammation and structural changes	
PsA	CASPAR	2006	Classification criteria	• PsA can be diagnosed in patients with a total score of ≥ 3 points across five domains (i.e., evidence of psoriasis, psoriatic nail dystrophy, RF negativity, dactylitis, juxta-articular bone formation)	[101]
				• PsA can manifest as symmetric polyarthritis, dactylitis-predominant disease, arthritis mutilans, or axial-predominant disease	
JIA, jSpA	ILAR	2001	Classification criteria	• JIA can be diagnosed in a patient with arthritis of unknown etiology that begins before the 16th birthday and persists for at least 6 weeks, excluding other known conditions	[111–113]
				• JIA can be classified into systemic arthritis, oligoarthritis, RF-negative polyarthritis, RF-positive polyarthritis, enthesitis-related arthritis, psoriatic arthritis, and undifferentiated arthritis	
	EULAR/PReS	2015	Points to consider	• MRI recommended for early detection, diagnosis, assessment, and monitoring, especially for TMJ and axial involvement	[137]
	ESSR Arthritis Subcommittee	2015	Recommendations	• MRI is recommended over clinical methods, XR or US for diagnostic confirmation, evaluation of challenging joints (e.g., hip, temporomandibular joint, subtalar joint, spine, sacroiliac joint), detection of subclinical disease foci, assessment of structural damage and complications, and treatment monitoring	[33]
	PRINTO	2019	Classification criteria	• JIA can be diagnosed in a patient with arthritis of unknown etiology that begins before the 18th birthday and persists for at least 6 weeks, excluding other known conditions	[58]
				• JIA can be classified into systemic JIA (a.k.a., Still disease), RF-positive JIA, enthesitis/spondylitis-related JIA, and early-onset ANA-positive JIA, other JIA, and unclassified JIA	
	ESSR-ESPR	2020	Recommendations	• MRI is recommended for detecting active inflammation in the axial skeleton, for confirming peripheral involvement, and for providing a baseline for disease monitoring	[119]
CAO	ESSR Arthritis Subcommittee	2018	Recommendations	• Whole-body MRI for detecting multifocal inflammation and disease monitoring	[41]

*RA* rheumatoid arthritis, *JIA* juvenile idiopathic arthritis, *axSpA* axial spondyloarthritis, *pSpA* peripheral spondyloarthritis, *PsA* psoriatic arthritis, *ReA* reactive arthritis, *EnA* enthesitis-related arthritis, *uSpA* undifferentiated spondyloarthritis, ACR* American College of Rheumatology, *ACR* American College of Radiology, *EULAR* European League Against Rheumatism, *PReS* Paediatric Rheumatology European Society, *ESSR* European Society of Skeletal Radiology, *ESPR* European Society of Paediatric Radiology, *ASAS* Assessment of SpondyloArthritis International Society, *OMERACT* Outcome Measures in Rheumatology, *CASPAR* ClASsification criteria for Psoriatic Arthritis, *ILAR* International League of Associations for Rheumatology, *PRINTO* Paediatric Rheumatology INternational Trials Organisation

To date, wbMRI has gained a central role in the detection, diagnosis, and monitoring of systemic musculoskeletal diseases by virtue of its comprehensive body coverage [4, 18]. Autoimmune arthritis represents the major non-oncologic musculoskeletal application domain of wbMRI, whereas the role reserved for non-autoimmune conditions such as crystalline, septic, and degenerative arthritis is virtually null. In adult patients, SpA represents the most established non-oncologic indication for wbMRI, whose protocol can be optimized according to the suspected pathologic entity [18]. In pediatric patients, rheumatologic disorders such as JIA and CRMO represent the most common indications for wbMRI along with lymphomas, metastatic disease, hereditary cancer syndromes, idiopathic pyrexia, and granulomatous illnesses [9, 17, 19, 51]. Nevertheless, reliable cost-effectiveness data thereupon remain scarce largely due to the technical heterogeneity of the published scan protocols, which prevented the development of consistent guidelines concerning the routine use of wbMRI in clinical rheumatological practice [17, 19]. For instance, neither the 2017 edition nor the 2022 update of the ACR appropriateness criteria for chronic extremity joint pain have brought wbMRI up among the provided recommendations [52, 53]. Moreover, although mentioning wbMRI for assessing the total disease burden, the 2021 update of the ACR appropriateness criteria for axial SpA does not provide any definite recommendation thereupon [54].

### Rheumatoid arthritis

Rheumatoid arthritis (RA) is characterized by autoimmune targeting of the synovium along with extraskeletal sites and affects around 1% of the adult population, with preference for females. The resulting synovial inflammation and pannus growth eventually lead to joint destruction, deformity, and chronic pain. Joint involvement in RA is typically polyarticular and symmetrical with early predilection for the small peripheral joints, while early involvement of larger joints is indicative of increased disease severity. RA can also affect the cervical spine, potentially causing instability or subluxation. Atypical presentations such as monoarticular arthritis or palindromic rheumatism may also occur [55–57]. Notably, the rare RF-positive polyarthritis encountered in childhood is considered the juvenile counterpart of adult RA [58] (Tables 1, 2).

The diagnosis of RA requires a high index of clinical suspicion based on the patient’s history, physical examination, and laboratory findings, also considering possible generalized symptoms, peri-arthritic syndromes, and extraskeletal manifestations. In fact, imaging findings need to be contextualized for avoiding misdiagnosis as, when taken alone, synovitis and other inflammatory signs are not specific to RA [55–57]. Although X-ray-based imaging can detect late structural abnormalities, its sensitivity to early signs of RA is scarce. Moreover, although US can detect synovial hyperemia, effusions, early erosions, impingement, subluxation, and tears, its low reproducibility, operator dependence, and shallow FoV substantially limit its diagnostic value [55, 57, 59]. By contrast, MRI excels in visualizing features of active RA and subsequent structural changes in the peripheral joints, allows reliable monitoring of treatment response, can identify concurrent inflammation in extra-articular sites, and enables (semi)quantitative assessment of inflammatory activity via DCE. MRI can be also conducted to evaluate the inflammatory status in the cervical spine and potential complications such as atlantoaxial subluxation, basilar invagination, and canal stenosis [13, 33, 56, 57, 60]. Prompt identification of RA is critical for achieving optimal response to treatment, which is witnessed by MRI evidence of suppression of joint inflammation. However, the clinical impact of MRI in the early diagnosis of RA is yet to be fully determined [13, 56, 60]. Also, even though MRI can surpass US in terms of diagnostic accuracy, the ultimate choice between the two techniques in RA remains substantially dependent upon the individual scenario [53]. Noteworthily, MRI evidence of bone edema can predict joint damage in early RA and enhance the predictive value of RF and CCP antibodies for RA development in undifferentiated arthritis [60]. The most utilized MRI-based scoring system for RA is RAMRIS, originally conceived in 1998 and updated in 2016 for hand MRI, although extendable to the foot [61, 62]. To this add the quicker RAMRIS-5, the simpler SAMIS, and the ERAMRS conceived for early RA [63].

**Fig. 3:**
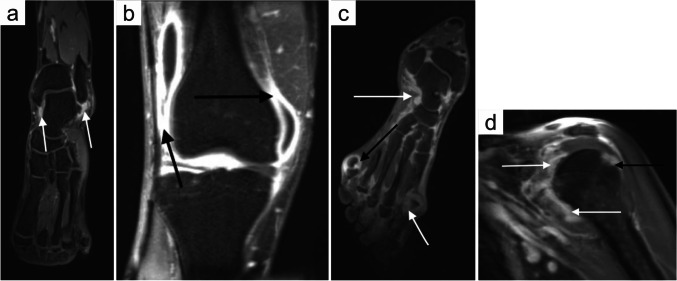
Post-contrast T1w VIBE Dixon water-only images from an abbreviated “multi-joint” MRI protocol (pseudowbMRI) in two patients with rheumatoid arthritis (**a–b** and **c–d**). **a** Oblique axial image of the ankle showing synovitis of the tibiotalar joint (white arrows); **b** Coronal image of the knee showing marked synovitis (black arrows) and joint effusion; **c** Oblique axial image of the foot showing synovitis (white arrows) and erosion (black arrows). **d** Coronal image of the shoulder showing extensive synovitis (white arrow) and erosion of the humeral head (black arrow) (reproduced from Freeston et al., 2024 [67], originally licensed under CC BY 4.0)

Although primarily affecting hands and feet, RA can potentially involve any joint, tendon, or enthesis in the body, which can be comprehensively captured via wbMRI. In fact, wbMRI can detect and assess the inflammatory burden in both axial and peripheral joints in a single scan and track the evolution of RA during treatment more reliably than clinical examination and US [18, 64, 65]. wbMRI and US agree and correlate well in terms of joint inflammation scores in clinically active RA before DMARD treatment at the patient level and, to a lesser extent, at the joint level and after treatment [13]. wbMRI was also shown to have good interscan agreement and very good intra- and inter-observer agreement in RA, although the agreement with conventional hand MRI varied likely due to different scoring systems and image quality [66]. Nonetheless, an abbreviated-wbMRI “multi-joint” protocol lasting less than 20 min has shown a potential for distinguishing among early RA, non-RA, and healthy controls [67] (Fig. 3). Although the current role of wbMRI in RA remains mostly investigational, promising reports about its diagnostic and monitoring performance do encourage larger validation studies for supporting its systematic implementation in clinical practice. Future research efforts on wbMRI in RA should be directed towards its early-diagnosis performance in at-risk individuals, optimization of scan coverage, and validation of quantitative disease-activity indices [18, 64, 65, 68–70].

### Seronegative spondyloarthropathy

“Seronegative spondyloarthropathy” (or spondyloarthritis) (SpA) is an umbrella term encompassing a heterogeneous spectrum of inflammatory conditions variably involving joints, syndesmoses, and extra-skeletal sites, hallmarked by enthesitis and negativity for the rheumatoid factor. Each SpA case can also be classified as either axial (axSpA) or peripheral (pSpA) according to the predominant distribution of disease manifestations. In adults, ankylosing spondylitis (AS) always manifests as axSpA, while most cases of psoriatic arthritis (PsA), reactive arthritis (ReA), enteropathic arthritis (EnA), and undifferentiated spondyloarthritis (uSpA) fall under the pSpA category. Juvenile SpA forms exhibit some differences from their adult counterparts and are discussed later in this article [71–73] (Tables 1, 2).

#### Axial spondyloarthropathy

axSpA is mostly represented by AS, a condition strongly associated with HLA-B27 and variably preferring male patients. This condition primarily affects the axial skeleton with a predilection for the sacroiliac joint, although peripheral sites can be secondarily involved [74–76]. axSpA is hallmarked by sacroiliitis, mainly manifesting as inflammatory back pain variably accompanied by peripheral arthritis and extra-articular manifestations, while isolated involvement of other skeletal sites is uncommon [73–76]. axSpA is conventionally defined as either non-radiographic (nr-axSpA) or radiographic (r-axSpA) according to the X-ray evidence of sacroiliac damage [71–73]. Noteworthily, such distinction is solely based on imaging, as the two entities do not substantially differ in most other features [73]. In r-axSpA, the inflammation primarily involving the lower, synovial portion of the sacroiliac joint leads to subchondral erosions and joint pseudo-widening, followed by bilateral sclerosis and ankylosis also visualizable via X-ray imaging. In the spine, inflammatory lesions in the annular entheses cause progressive vertebral squaring and syndesmophytosis until ankylosis, resulting in the classic “bamboo spine” [77, 78]. By contrast, nr-axSpA patients lack radiographic signs of sacroiliitis but meet clinical criteria for axSpA diagnosis, with MRI often showing signs of active sacroiliac inflammation. As ~ 10% of these patients develop radiographic structural changes within 2 years, nr-axSpA is considered prodromic to r-axSpA [73]. Although specific for axSpA, spinal lesions contribute little to its classification as they rarely occur without imaging evidence of sacroiliitis [72, 77] (Tables 1, 2).

According to the 2009 ASAS classification criteria, axSpA can be diagnosed in patients younger than 45 years with chronic back pain following either the “clinical arm” (i.e., positivity to HLA-B27 +  ≥ 2 other SpA clinical features) or the “imaging arm” (i.e., imaging evidence of sacroiliitis +  ≥ 1 SpA clinical feature) [73, 79]. MRI has revolutionized the management of axSpA by enabling earlier diagnosis and treatment [5]. Even though XR remains the first-line investigation for detecting structural changes in the long-standing disease, MRI is superior for detecting early sacroiliitis when radiographic abnormalities are still absent [73, 79]. In fact, the inclusion of sacroiliac MRI in the imaging arm has led to the latter accounting for ~80% of nr-axSpA diagnoses [74, 80–82]. Consequently, MRI has become the imaging modality of choice for axSpA by virtue of its high sensitivity for pre-radiographic signs of active axial inflammation. In addition, later radiographic structural changes, as well as additional phenomena such as fat metaplasia, tissue backfill, tenosynovitis, and aseptic spondylodiscitis, can all be detected via MRI [18, 65, 74]. However, as bone marrow edema is an unspecific finding, the combination with clinical evidence is required to increase the specificity of MRI findings and minimize misdiagnosis [75, 83, 84]. In ground-truth clinical practice, the imaging pathway in axSpA includes sacroiliac XR (according to the 1984 modified New York radiologic criteria) and MRI as well as spine wide-FoV MRI including the costovertebral and costotransverse joints, which challenge XR due to rib overlaying and radiation concerns [85]. In addition to conventional fluid-sensitive and anatomic sequences, DWI/ADC and GBCA could further improve the accuracy of MRI in axSpA through better detection of osteitis compared to bone marrow edema alone, delayed gadolinium-enhanced MRI of cartilage (dGEMRIC), and (semi)quantitative assessment of inflammation via DCE [74]. MRI-based scoring systems for axSpA include ASspiMRI-a and -c, the Berlin method, SPARCC, and the Canada-Denmark (CANDEN) system [86, 87].

**Fig. 4:**
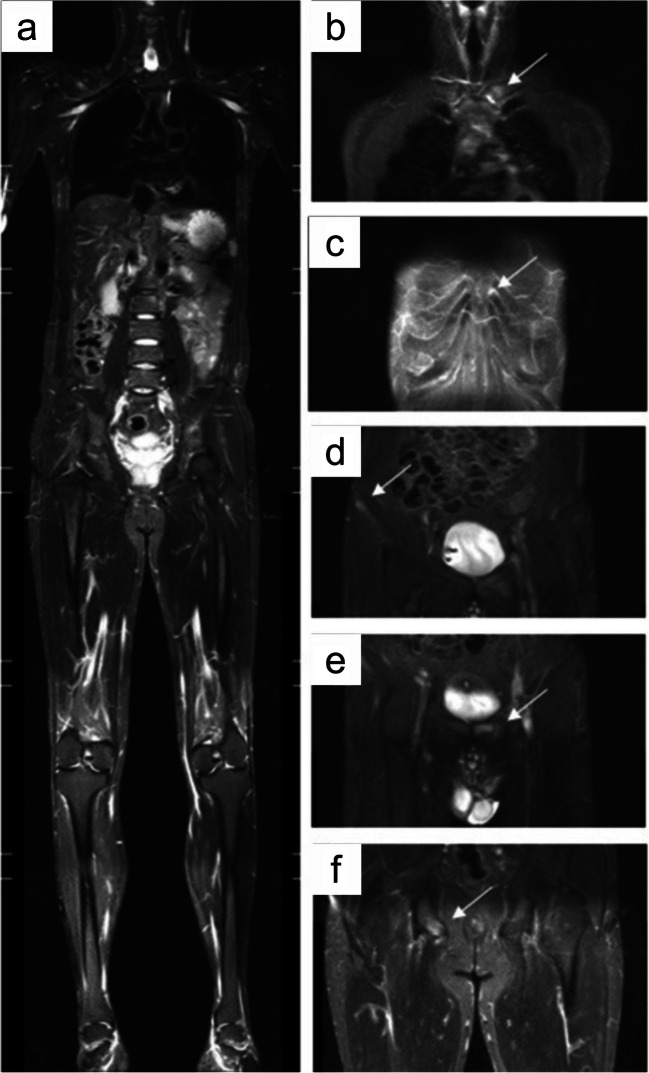
wbMRI of a 36-year-old, HLA-B27-positive man diagnosed with ankylosing spondylitis. **a** Coronal shoulder-to-heel STIR image;** b** Enthesitis of the left sternoclavicular joint (white arrow); **c** Enthesitis of the left costosternal joint (white arrow); **d** Enthesitis of the right anterior superior iliac spine (white arrow); **e** Enthesitis of the pubic symphysis (white arrow); **f** Enthesitis of the right ischial tuberosity (white arrow) (adapted from Guo et al., 2022 [72], originally licensed under CC BY 4.0)

wbMRI bolsters clinical diagnosis of axSpA through valuable imaging information concerning disease distribution, inflammatory activity, and established joint damage [33]. wbMRI can distinguish between active inflammation and structural changes in all symptomatic axial and peripheral sites at once, eventually detecting additional subclinical disease foci in challenging sites such as the thoracic spine, ultimately increasing diagnostic reliability [4, 23]. Concurrent wbMRI evidence of enthesitis at the sacroiliac joints, vertebral corners, zygapophyses, and/or proximal costal joints, seldom accompanied by dactylitis, is strongly suggestive of axSpA and can be utilized for monitoring treatment response [75]. wbMRI also allows imaging of anterior chest-wall sites, often bypassed in conventional MRI studies, although being impacted in almost half of axSpA patients [4, 76] (Fig. 4). Importantly, peripheral entheseal involvement can be documented in up to three-quarters of axSpA cases, possibly even in patients without sacroiliitis, reinforcing the potential diagnostic role of wbMRI [4, 23, 88, 89]. Peripheral enthesitis holds variable diagnostic value for axSpA according to the involved site, further warranting wbMRI in at-risk individuals. Enthesitis scoring may offer a more accurate diagnosis than simple site counting, also considering its different distribution between axSpA and pSpA, keeping in mind that abnormal entheseal signals should be contextualized to prevent misdiagnosis [72]. wbMRI has demonstrated a good correlation to conventional MRI for bone marrow edema in the sacroiliac joint, while the same aspect in the spine was more variable [68]. In addition, wbMRI can objectively demonstrate a reduction in inflammation in axSpA following DMARD treatment [68, 75]. All these aspects seem to justify wbMRI as a reliable tool for the diagnosis and monitoring of axSpA, although its actual prognostic value needs further elucidation [75, 90].

#### Peripheral spondyloarthropathy

pSpA forms are characterized by variable involvement of peripheral joints and entheses with minor and/or later impact on the axial skeleton. Among these, PsA often follows an asymmetric mono- or oligoarticular pattern, hallmarked by enthesitis-related dactylitis with diaphyseal osteitis and predominant involvement of the flexor tendons (vs. extensor tendons in RA). However, skeletal involvement in PsA is highly variable and possibly changing over time, possibly including symmetric polyarthritis, dactylitis-predominant disease, arthritis mutilans, or axial-predominant disease, even before skin manifestations (i.e., “psoriatic arthritis sine psoriasis”) [77, 91–94]. Reactive arthritis (ReA) (a.k.a., Reiter’s syndrome) generally follows a urogenital or gastroenteric infection, tends to chronicize, and often presents as an asymmetric oligoarticular arthritis predominantly involving the large joints of the lower extremities. This is possibly accompanied by enthesitis, bursitis, tenosynovitis, upper-extremity involvement, and, less commonly, axial involvement [73, 77]. Enteropathic arthritis (EnA) is associated with several chronic bowel illnesses, mainly inflammatory bowel disease (IBD), and presents either as peripheral oligoarthritis or polyarthritis with possible axial involvement, although no formal diagnostic criteria have been proposed [95, 96]. Undifferentiated spondyloarthritis (uSpA) is a debated entity encompassing all those forms not fully satisfying the established classification criteria for other SpA subtypes [97, 98] (Tables 1, 2).

According to the 2011 ASAS classification criteria, patients with peripheral-only manifestations of arthritis, enthesitis, and/or dactylitis demonstrating other general SpA features are diagnosed with pSpA [99]. Imaging assessment of pSpA focuses on peripheral synovial spaces, soft tissues, bony surfaces, and particularly entheses, whose inflammation often challenges clinical diagnostic methods [14]. Even though XR and US do offer several advantages in terms of simplicity and availability, MRI boasts higher diagnostic earliness, reliability, and reproducibility for pSpA, being capable of objectively assessing inflammatory status, structural changes, and disease progression as well as to clarify doubtful axial findings [100]. The specific diagnosis of PsA is generally confirmed via the 2006 CASPAR criteria, with XR capable of revealing erosive bone changes and new bone formation and US capable of detecting active inflammation [77, 92, 93, 101]. Nevertheless, MRI in PsA can objectively assess inflammation of both the fibrous and bony parts of entheses and surrounding tissues and can also detect subclinical inflammation foci in patients with psoriasis, possibly predictive of future PsA development [14, 33, 91, 93]. As in RA, correlation of MRI-detected bone marrow edema with erosions and deformity in peripheral sites has been demonstrated in PsA. In addition, typical signs of axial inflammation and structural damage in PsA are well depicted by MRI, typically at the vertebral corners and endplates, proximal costal joints, and sacroiliac joints [91, 102]. Concerning ReA, post-urethritis and HLA-B27-positive ReA cases exhibit more severe MRI features than RA and post-enteritis ReA [103]. In EnA, MRI can detect arthritic manifestations even before the clinical evidence of IBD [104] and, vice versa, enterography MRI scans can detect asymptomatic sacroiliitis in young patients investigated for IBD. Therefore, MRI might anticipate the diagnosis of EnA even in the subclinical patient [36].

The OMERACT MRI in Enthesitis Initiative proposed the heel-based scoring system HEMRIS for standardizing the assessment of peripheral enthesitis in pSpA [100]. A further scoring system named PsAMRIS has been conceived for specific application to the hands and forefeet of PsA patients [91]. An even more relevant score system not restricted to a specific diagnosis is MRI-WIPE, conceived for comprehensive whole-body assessment of peripheral joints and entheses. In MRI-WIPE, arthritis and enthesitis are assessed according to previously proposed definitions for, on the one hand, synovitis and osteitis at joints and, on the other hand, soft tissue inflammation and osteitis at entheseal sites. Overall, 83 peripheral joints and 33 entheses are assessed and their components scored for inflammatory involvement from 0 to 3 each. The final MRI-WIPE score is calculated by summing up all scores from joints (0–537) and entheses (0–201), yielding a total score range of 0–738 [42, 46, 47].

**Fig. 5:**
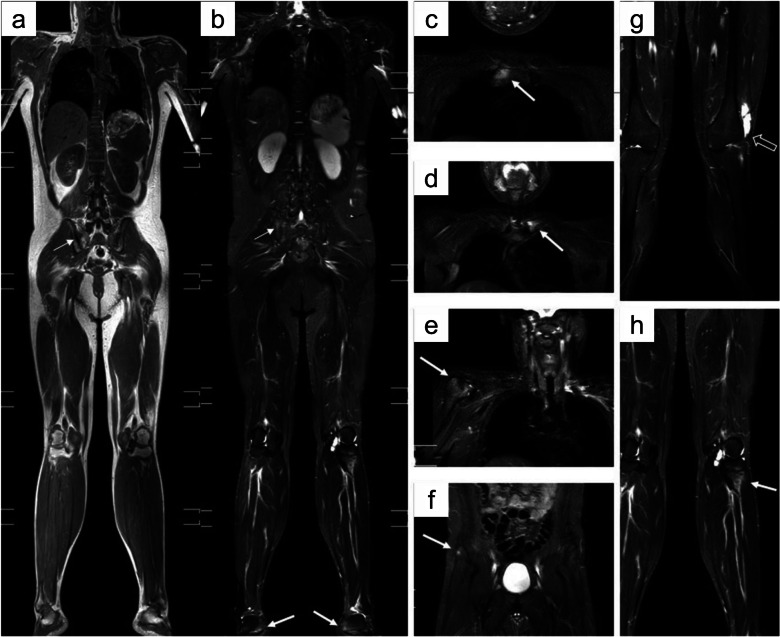
wbMRI of an 18-year-old male SpA patient with both axial and peripheral manifestations. **a**, **b** Coronal T1w and STIR shoulder-to-heel images showing sacroiliitis (thin white arrow) and bone marrow edema in both heels (white arrow). **c–h** STIR images of the sternoclavicular region, greater tuberosity of the humerus, pelvis, and knees showing multiple foci of bone marrow edema (white arrows) and left knee effusion (hollow arrow) (reproduced from Guo et al., 2021 [139], originally licensed under CC BY-NC-ND 4.0)

As anticipated, inflammation in pSpA can be widespread and variable, especially in PsA, often challenging to capture using conventional small-FoV MRI, also considering the possibility of subclinical foci as well as axial involvement [14, 105] (Fig. 5). As for other autoimmune arthritides, wbMRI can aid in diagnosing and assessing treatment response in PsA by identifying, quantifying, and monitoring juxta-articular inflammation and bone damage. The ability to image all tenosynovial sheaths and entheses within the body makes wbMRI a promising and reproducible “one-stop-shop” imaging modality in PsA [18, 65]. The hands and feet deserve special attention in PsA, needing to be carefully included in the FoV and sometimes also scanned via dedicated acquisitions. In fact, routine assessment of distal extremities in whole-body acquisitions is hampered by limited image quality in wbMRI, although technical advancements might overcome such an issue in the near future [14]. wbMRI in PsA has demonstrated good patient tolerance and high sensitivity for subclinical inflammation, with moderate agreement between enthesitis on clinical scores. The possibility of wbMRI to evaluate the total inflammation burden in both axial and peripheral joints and entheses is likely to increase diagnostic confidence and earliness [106–108]. Many uSpA cases are known to possibly transition towards a more definite SpA form, likely due to the frequently low specificity of disease patterns in the early SpA stages. The inclusion of wbMRI in the initial diagnostic workup of uSpA cases could anticipate the disclosure of a more specific entity, as previously demonstrated [109].

### Juvenile idiopathic arthritis

The term “juvenile idiopathic arthritis” (JIA) is an exclusion diagnosis with a prevalence of roughly 0.1% in the general population, whose classification system is currently undergoing thorough renewal [58, 110]. The 2001 ILAR classification distinguishes JIA into six different entities plus an additional undifferentiated category [111–113]. However, the heterogeneity and overlapping of some categories and the overlook of axial involvement have triggered interest in renovating the ILAR classification, as attempted by PRINTO in 2019 [58, 114, 115]. Noteworthily, while all JIA subtypes exhibit synovial inflammation, the PRINTO “enthesitis/spondylitis-related JIA” category emphasizes entheseal inflammation, covering most ILAR’s ERA and juvenile-onset ASAS’ SpA cases [110, 116]. Although intersocietal consensus upon JIA definitions and classification is still necessary, the latter category could be referred to as “juvenile SpA” (jSpA) [117]. While most JIA cases presumably represent the adult counterparts of systemic, rheumatoid, and undifferentiated arthritides while also including early-onset ANA-positive JIA, about 20% are represented by jSpA [22, 58, 114, 117]. In non-jSpA JIA, localized synovial hyperemia and pannus proliferation in peripheral joints can impact epiphyseal and physeal cartilages, resulting in epiphyseal overgrowth, premature ossification, and skeletal deformities, to which often adds the involvement of the temporomandibular joint and cervical spine [118]. On the other hand, jSpA primarily affects the entheses of male children, with a preference for the sacroiliac joints and thoracolumbar spine and, in the peripheral skeleton, the patella, greater trochanter, calcaneus, and tarsus [22, 114, 117] (Tables 1, 2).

Non-jSpA JIA and jSpA respectively exhibit preferential involvement for the peripheral and axial skeleton, although overlapping is frequent, particularly in later phases [110, 119]. However, the evaluation of pediatric rheumatic disorders differs from that in adults in several aspects. A key challenge in childhood imaging is represented by early diagnosis, critical to start treatment during the “window of opportunity,” ultimately favoring remission and prognosis [15, 110]. Also, radiologists’ familiarity with the normal osteochondral and intramedullary development on the skeleton is a critical requirement for retaining optimal accuracy in JIA diagnosis [15, 110]. Noteworthily, residual hematopoietic marrow in the feet of healthy children might determine edema-like changes possibly mimicking asymptomatic early inflammation, although being usually symmetrical and disappearing with age [22, 114].

Although XR and US also have a relevant role in JIA, MRI excels in the early assessment of inflammatory arthritis and enthesitis much earlier than structural damage [110, 119]. In fact, MRI can assess all the musculoskeletal structures potentially involved in JIA by virtue of its multiplanar capabilities and excellent detail and contrast of soft tissue, making it very sensitive to early signs of soft tissue inflammation and osteochondral damage. The absence of ionizing radiation is the most valuable advantage of MRI in pediatric cases, although the frequent need for sedation in younger patients limits its use in everyday practice [110]. While early peripheral involvement in JIA is also examinable via US, early axial involvement is mostly oligo- or asymptomatic and detectable only via MRI [22, 114, 116, 119]. Also, DCE MRI might help to differentiate among active inflammation, inactive inflammation, and physiological growth-related joint hyperemia in JIA [119]. MRI scoring systems commonly used in adult autoimmune arthritis do not necessarily preserve reliability in children, although childhood-specific systems have already been proposed (e.g., JAMRIS) [119]. A multitude of initiatives have been organized to promote research and optimization regarding the use of imaging in JIA [120].

**Fig. 6:**
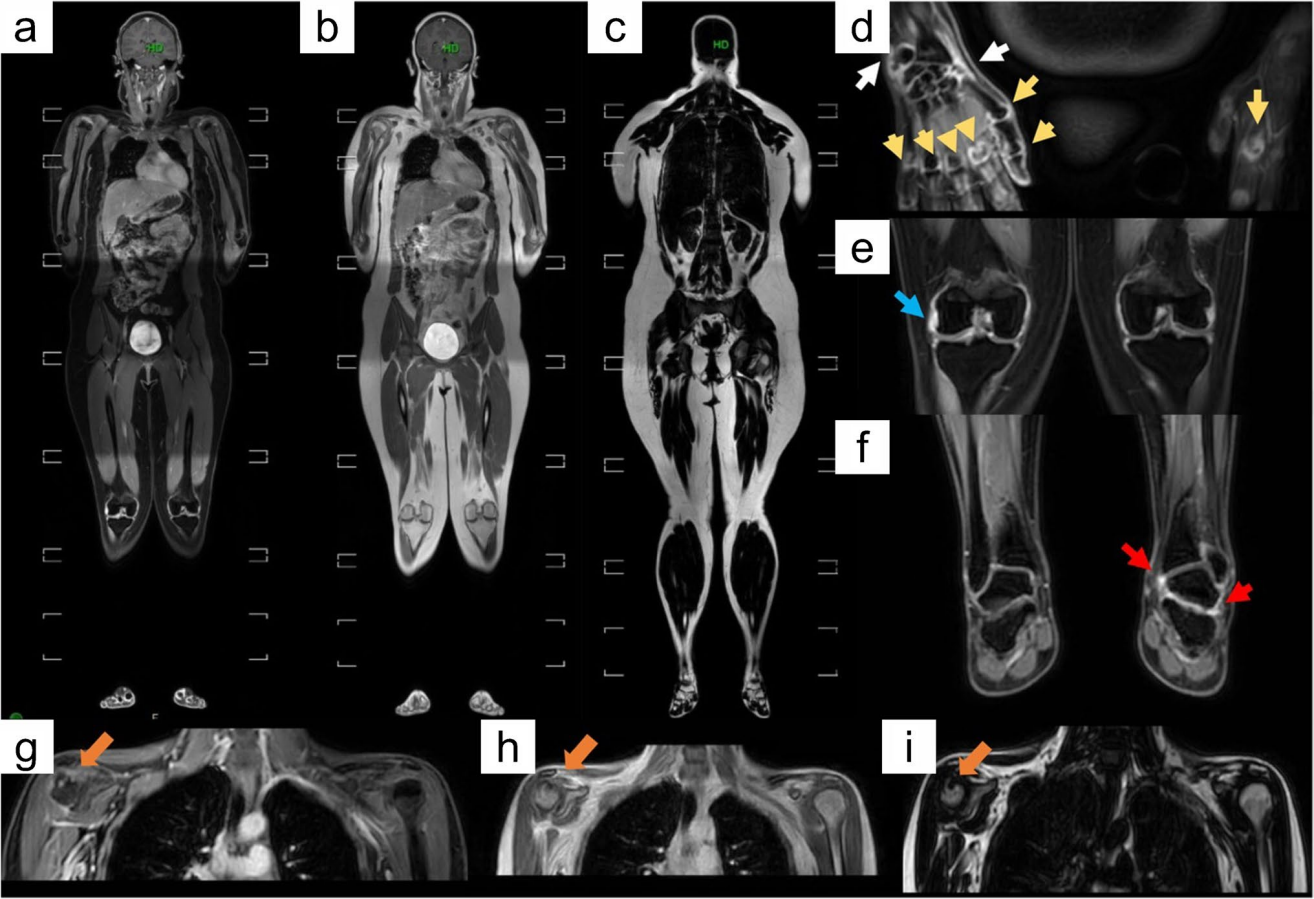
Post-contrast wbMRI images of two patients with juvenile idiopathic arthritis (**a–f** and **g–i**). **a** Vertex-to-heels Dixon water-only image; **b** Vertex-to-heels Dixon in-phase image; **c** Vertex-to-heels Dixon fat-only image; **d** Grade-2 synovitis of the right wrist (white arrows), right 1st–5th metacarpophalangeal, right 1st interphalangeal, and left 2nd metacarpophalangeal joints (yellow arrows and arrowheads); **e** Grade-2 synovitis of the right knee (blue arrow); **f** Grade-2 synovitis of the left tibiotalar and subtalar joints (red arrows). **g–i** Erosion at right glenohumeral joint (orange arrows) (reproduced from Choida et al., 2024 [140], originally licensed under CC BY 4.0)

wbMRI currently boasts a diverse range of clinical pediatric applications, including the assessment of overall skeletal maturity and neoplastic or non-neoplastic conditions. Due to its comprehensive coverage and absence of ionizing radiation, wbMRI is also emerging as the preferred initial imaging modality for early diagnosis, staging, and treatment response evaluation in juvenile rheumatic diseases [18, 65]. In JIA, wbMRI is being increasingly utilized for quantifying the inflammatory burden in the peripheral and axial skeleton and for guiding treatment decisions, particularly by virtue of the absence of ionizing-radiation exposure, overall reduction of examinations under sedation or anesthesia, and high tissue-contrast resolution [15, 22, 110, 118] (Fig. 6). wbMRI can detect and map active inflammation foci and structural changes in JIA with good correlation with US and more objectively and comprehensively than clinical methods, which is particularly valuable when the patient is too young to communicate symptoms [22, 110, 118, 121, 122]. Despite its advantages, the high costs, limited accessibility, and long acquisition times of wbMRI limit its applicability in pediatric settings. Also, whole-body acquisitions struggle to distinguish fluid from synovitis without GBCA administration, to differentiate among the various cartilage subtypes in the immature skeleton, and to assess oblique joint morphology and small pathologic abnormalities. Therefore, wbMRI-based scoring systems may lose reliability in pediatric patients unless complemented by targeted smaller-FoV acquisitions [15, 118]. In addition, age-related differences in skeletal morphology, physis closure stages, and red marrow distribution challenge the interpretation of wbMRI, ultimately decreasing its specificity. This aspect can nonetheless be mitigated by expertise in pediatric musculoskeletal radiology and utilization of MRI atlases [110, 118]. In contrast to adult rheumatisms, clear guidelines concerning the utilization of wbMRI in non-jSpA JIA and jSpA are still lacking [22, 110, 118], although a roadmap for future investigation on the role of wbMRI in this context has been proposed [15].

### Chronic aseptic osteitis

The SAPHO syndrome is a rare chronic-relapsing inflammatory disorder of young adults of unknown etiology characterized by aseptic synovitis, acne, pustulosis, hyperostosis, and osteitis. Skeletal manifestations can be either preceded or followed by skin manifestations, often with a lag of several years. Some “incomplete” forms of SAPHO have been reported, including sternocostoclavicular hyperostosis (SCCH) and pustulotic arthro-osteitis (PAO, a.k.a. Sonozaki syndrome). Chronic nonbacterial osteomyelitis (CNO) refers to idiopathic bone inflammation mostly affecting children, which upgrades to chronic recurrent multifocal osteomyelitis (CRMO) when featuring recurrent multifocal inflammatory flares. CRMO is often considered the pediatric analog of SAPHO, although with differences in preferential skeletal sites [123–126]. Common axial involvement, frequent association with inflammatory bowel disease and psoriasis, and clinical-radiological overlap support the interrelation among SAPHO, CRMO, and SpA [9, 17, 110]. Although definitive evidence thereupon is still lacking [6], SAPHO and CRMO will be discussed altogether in this section under the umbrella term of “chronic aseptic osteitis” (CAO) (Tables 1, 2).

Musculoskeletal manifestations of CAO encompass age-dependent, localized or multifocal, sterile osteitis commonly affecting the thoracic and spinopelvic bones in adults, while preferring long tubular bones in children. Osteitis foci can be synchronous or metachronous, possibly subclinical, and generally involving both the cortical and medullary perimetaphyseal bone, associated with endosteal and periosteal new bone formation and entheseal sclerosis and hyperostosis. Further phenomena include juxtaphyseal nodules, periosseous edema, myositis, and synovitis [6]. Arthritis in CAO is generally oligoarticular and asymmetric with a preference for the large joints of the lower limb, although small joints of the hands and feet can also be involved. In advanced disease, peripheral enthesopathy worsens towards deformity, to which can add spondylodiscitis, asymmetrical hyperostosis, and ankylosis at the sacroiliac joints and multiple vertebral levels. In some cases, vertebral collapse and subsequent deformity may occur, potentially leading to misdiagnosis as a neoplasm or infection [127].

The diagnosis of CAO relies primarily on clinical presentation supported by laboratory, radiological, and histological findings, yet is often delayed, with most patients consulting with up to five physicians and undergoing multiple segmental examinations before receiving a definitive diagnosis. This can be even more challenging in the absence of skin lesions, atypical disease distribution, or oligosymptomatic manifestations [6, 123, 127]. While X-ray-based imaging mostly focuses on structural abnormalities, MRI can reveal both active and chronic arthritic features in CAO, such as vertebral corner lesions, aseptic spondylodiscitis, prevertebral soft tissue edema, and PsA-like asymmetric syndesmophytosis, as well as late complications such as vertebral fractures [6]. Axial involvement in CAO can be asymptomatic in some cases, highlighting the utility of thoracolumbar spine MRI for early detection [127]. The concurrent presence of multifocal osteitis and synovitis in children has been recentlydescribed as “CRMO–JIA overlapping syndrome” [128].

**Fig. 7:**
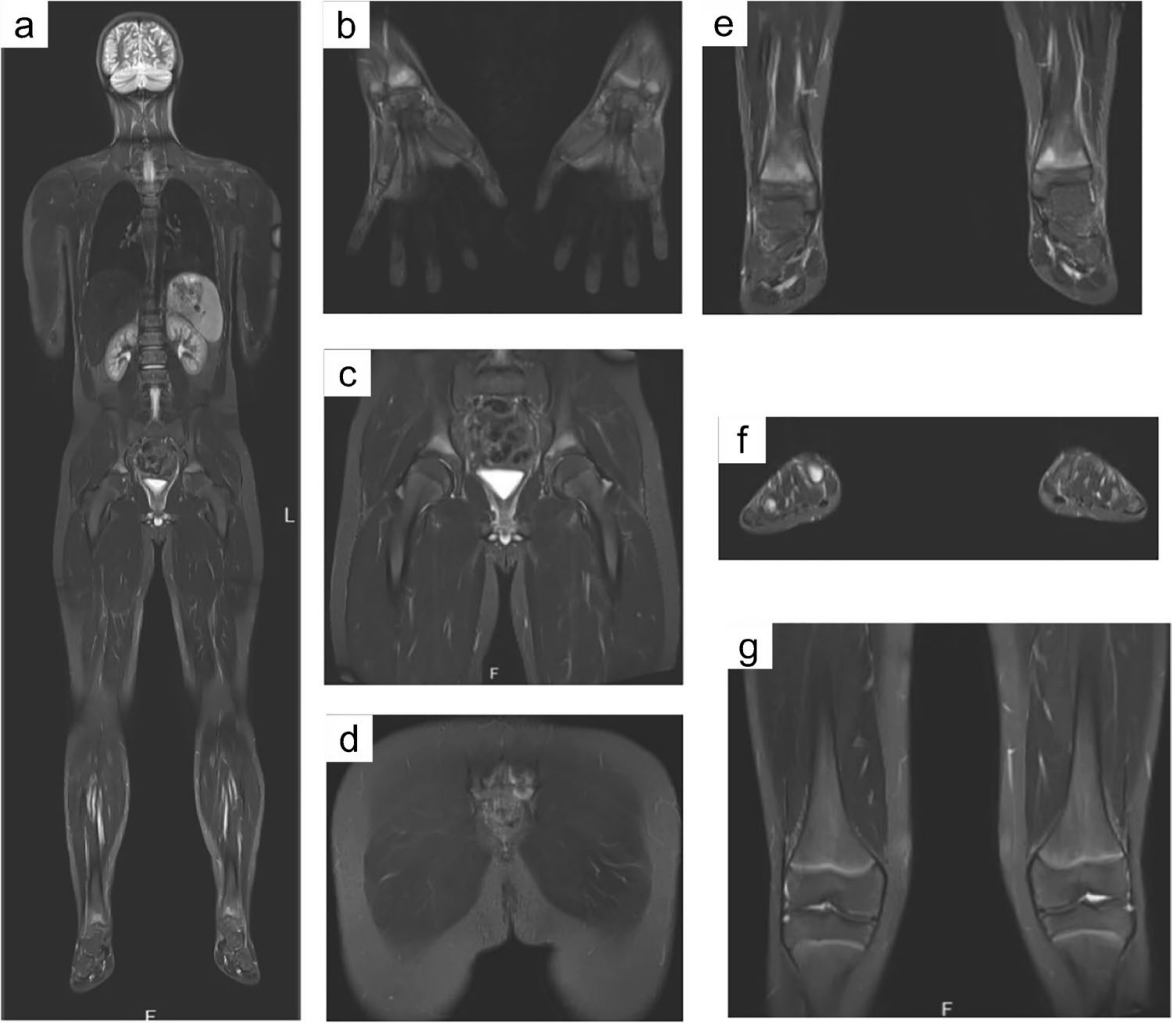
wbMRI of a 12-year-old male patient with CRMO. **a** Coronal vertex-to-heels TIRM image; **b–g** Zoomed images demonstrating symmetrical inflammatory bone lesions of the distal radii (**b**), iliac bones (**c**), sacrum (**d**), distal tibiae (**e**), and metatarsal bones (**f**), with no involvement of the knees (**g**) (reproduced from Hedrich et al., 2020 [141], originally licensed under CC BY 4.0)

The multifocal and relapsing–remitting nature of the disease and the frequent involvement of the axial skeleton justify the utilization of wbMRI in CAO, which is convenient for anticipating diagnosis and monitoring disease progression, particularly valuable in children (Fig. 7). Notably, wbMRI can discover almost 50% more lesions than clinical examination [6, 51]. Currently, wbMRI is the gold standard imaging modality in CAO, as it offers a comprehensive approach for identifying and monitoring arthritis, enthesitis, and osteitis in axial and peripheral symptomatic or asymptomatic sites. In addition, concurrent phenomena such as myositis and fasciitis can also be comprehensively characterized and monitored via wbMRI [9, 127, 128].

## Whole-body MRI scan protocols

Fluid-sensitive sequences like STIR are the most utilized “core” whole-body acquisitions in adults as in children by virtue of their excellent sensitivity for active inflammation, followed by T1w sequences providing anatomical detail [5]. In addition, SS EPI DWI with ADC mapping, Dixon, and post-GBCA acquisitions with DCE can be electively performed for improving visualization and assessment of active inflammatory foci [5, 129–131]. Any core wbMRI protocol may also require ancillary, smaller-FoV sequences according to the clinical scenario, technical stage of the MRI unit, and local circumstances, particularly for smaller joints and younger patients [5, 14]. The total scan duration can also exceed 60 minutes, yet it could be decreased by truncating the coverage of the whole-body acquisition, which should include the upper limbs with the hands placed above the belly or behind the lower back [23, 24]. A general wbMRI scan protocol for non-oncologic musculoskeletal applications based on various available recommendations and suggestions [9, 16, 22, 33, 41, 132] is presented in Table 3.

**Table 3:** General wbMRI scan protocol for musculoskeletal rheumatologic applications in an adult patient, with indicative values as if performed in a 1.5 T MRI unit (partially based on information from [9, 16, 22, 24, 33, 41, 132])

#	Sequence type	Coverage	Plane	Role(s)	Stations	T_E_/T_R_/T_I_ (ms)	ETL	NEX	FoV per station (mm)	Matrix (ν × φ)	Slice thickness/slice gap (mm)	Number of slices	Average scan time (min)
1	3D T1w VIBE Dixon	Whole body	Coronal	Anatomic detail	5–7	2.5/4.2/-	1	1	500	384 × 269	5/1	35	5
2	STIR	Whole body	Coronal	Inflammatory overview	5–7	60/4000/160	18	1	500	384 × 269	5/1	35	35
3	3D T1w VIBE Dixon	Whole spine	Sagittal	Anatomic detail	2–3	2.5/4.2/-	1	1	400	512 × 256	4/0.4	15	3
4	STIR	Whole spine	Sagittal	Inflammatory overview	2–3	60/4000/160	18	1	400	512 × 256	4/0.4	15	15
±	DWI/ADC*	Whole body	Coronal	Inflammatory overview (alternative), hypercellularity	5–7	70/3000/-	1	3	500	384 × 269	5/1	35	20
±	STIR	Targeted	Oblique	Detailing of unclear inflammatory foci	1	60/4000/160	18	2	180	256 × 192	5/0.5	15	12
±	3D T1w VIBE Dixon	Targeted	Oblique	Detailing of unclear structural changes	1	2.5/4.2/-	1	1	180	256 × 192	3/0.3	20	2
±	PDw TSE FS	Targeted	Oblique	Cartilage assessment	1	30/2000/-	10	1	180	256 × 192	5/0.5	15	4
±	Others**	Regional	Various	Various**	1–2	Various	Various	Various	Various	Various	Various	Various	Various
±	3D T1w VIBE Dixon, GBCA + ***	As **1** or **3**	As **1** or **3**	Enhancement of hypervascular tissues	As **1** or **3**	As **1** or **3**	As **1** or **3**	As **1** or **3**	As **1** or **3**	As **1** or **3**	As **1** or **3**	As **1** or **3**	As **1** or **3**

*NEX* number of excitations, *ν* frequency, *φ* phase, *T*_*E*_ echo time, *T*_*R*_ repetition time, *T*_*I*_ inversion time

TIRM, T2w TSE FS, or DWI could be used alternatively to STIR; T1w SE or TSE could be used alternatively to 3D T1w VIBE Dixon

^*^Useful *b*-values in s/mm^2^ include 0, 50, 600, and > 1000 (synthetic)

^**^e.g., SSFP T2*w GRE for magnetic susceptibility, solid organs, bowel, or lungs; UTE/ZTE for lungs, compact bone, or connective tissues; FLAIR for the central nervous system

^***^With or without DCE and dGEMRIC

In adults, a wbMRI protocol for RA should also include bi- or triplanar anatomic and fluid-sensitive acquisitions of the upper cervical spine [33]. A wbMRI scan protocol specific for axSpA should include supplementary coronal oblique and axial oblique anatomical, fluid-sensitive, and cartilage-oriented acquisitions of the sacroiliac joints and, if needed, of affected spine segments [4, 33]. For pSpA, targeted small-FoV anatomical and fluid-sensitive acquisitions of suspected sites should be performed, although a “screening” oblique sagittal fluid-sensitive acquisition of the hindfoot has also been proposed [24]. Any extraskeletal finding (e.g., muscles, brain, bowel, lungs, soft tissues) should be further investigated as possibly indicating a another non-primarily musculoskeletal diagnosis, [51].

In children, STIR is the main sequence in wbMRI protocols for JIA, as seen for adults [22], although DWI seems to perform better in JIA compared to T2w and STIR sequences [133]. Assessment of the temporomandibular joint in suspect JIA can be challenging and requires small-FoV open- and closed-mouth acquisitions in the coronal and sagittal planes [118, 119]. Involvement of the patellar and calcaneal entheses in jSpA requires targeted small-FoV acquisitions in the sagittal plane [118, 119]. A STIR-only wbMRI scan protocol for jSpA has been proposed, which includes a core whole-body scan in the coronal plane supplemented by sagittal scans for the spine, knees, and ankles, an axial scan for the pelvis, and a coronal oblique scan for the sacroiliac joints [9]. The core wbMRI protocol (i.e., STIR and T1w whole-body and whole-spine acquisitions) is considered sufficient for the evaluation of SAPHO/CRMO [123].

## Conclusion

In conclusion, wbMRI is a versatile and efficient tool for assessing inflammatory arthritides, offering a comprehensive overview of the body tailorable to the available clinical, laboratory, or imaging information. In addition to the general advantages of MRI technology, such as high tissue contrast, safety, and reproducibility, the major strengths of wbMRI include its easy access to the axial skeleton and its large FoV encompassing the whole peripheral skeleton. As early and accurate diagnosis is crucial for improving the prognosis of most inflammatory arthritides, the ability of wbMRI to depict the entire disease distribution and to detect subclinical foci is of paramount importance. While wbMRI does not have a relevant role in non-autoimmune arthritides, its diagnostic convenience appears substantial in RA and even critical in seronegative conditions such as SpA, JIA, and CAO. Integration of clinical findings and laboratory parameters further increases the overall diagnostic value of wbMRI, in line with the latest societal guidelines. The use of standardized assessment tools such as MRI-WIPE during wbMRI reporting is encouraged for a more reliable assessment of treatment response during follow-up. A key factor for effectively integrating wbMRI in local imaging protocols is scan time optimization, achievable via specialized equipment, advanced sequences, and case-tailored coverage. Here we proposed a general “core” wbMRI protocol for inflammatory arthritis, to be complemented by regional and/or targeted acquisitions according to the clinical scenario.

## Data Availability

Not applicable.

## Abbreviations

ADC: Apparent diffusion coefficient
ASAS: Assessment of SpondyloArthritis International Society
CAO: Chronic aseptic osteitis
CHESS: Chemical-shift spectral saturation
CISS: Constructive interference in steady state
CAO: Chronic aseptic osteitis
CNO: Chronic nonbacterial osteomyelitis
CRMO: Chronic recurrent multifocal osteomyelitis
DCE: Dynamic contrast enhancement
DESS: Dual-echo steady state
DMARD: Disease-modifying anti-rheumatic drug
DWI: Diffusion-weighted imaging
DWIBS: Whole-body DWI with background suppression
EPI: Echo-planar imaging
ESSR: European Society of Skeletal Radiology
FISP: Fast magnetic resonance imaging in steady-state precession
FLAIR: Fluid-attenuated inversion recovery
FoV: Field of view
FS: Fat-saturated
GBCA: Gadolinium-based contrast agents
GRE: Gradient-recalled echo
HASTE: Half-Fourier acquisition single-shot turbo spin-echo imaging
HEMRIS: Hip Enthesitis Magnetic Resonance Imaging Scoring
JAMRIS: Juvenile Arthritis Magnetic Resonance Imaging Scoring
JIA: Juvenile idiopathic arthritis
MPR: Multiplanar reformation
MRI-WIPE: Magnetic Resonance Imaging Whole-body Inflammation and Proliferation Evaluation
OMERACT: Outcome Measures in Rheumatology
PsAMRIS: Psoriatic Arthritis Magnetic Resonance Imaging Scoring
PSIF: Time-reversed FISP
RA: Rheumatoid arthritis
RAMRIS: Rheumatoid Arthritis Magnetic Resonance Imaging Scoring
SAPHO: Synovitis, acne, pustulosis, hyperostosis, and osteitis
SE: Spin-echo
SNR: Signal-to-noise ratio
SpA: Spondyloarthropathy
SS: Single-shot
SSFP: Steady-state free precession
STIR: Short inversion time inversion recovery
T1w: T1-weighted
T2*w: T2*-weighted
T2w: T2-weighted
TIRM: Turbo inversion recovery magnitude
TSE: Turbo spin-echo
USPIO: Ultra-small superparamagnetic iron oxide
UTE: Ultra-short echo time
VIBE: Volumetric interpolated breath-hold examination
wbMRI: Whole-body magnetic resonance imaging
ZTE: Zero echo time
